# The influence of air pollution on pollen allergy sufferers 

**DOI:** 10.5414/ALX02284E

**Published:** 2021-12-01

**Authors:** Markus Berger, Maximilian Bastl, Johannes Bouchal, Lukas Dirr, Uwe Berger

**Affiliations:** 1 Institute of Pathophysiology and Allergy Research, Center for Pathophysiology, Infectiology and Immunology, and; 2 University Department of Otolaryngology, Medical University of Vienna, Austria

**Keywords:** air pollution, ozone, symptom data

## Abstract

A multitude of consequences from global warming and environmental pollution can already be seen for nature and humans. The continuous burning of fossil fuels leads to rising temperatures and rising water levels causing extreme weather phenomena like heat waves and flooding. Increasing levels of air pollution also cause adverse health effects. This is especially important for pollen allergy sufferers because air pollution plays a central role in the interactions between pollen and humans. Today, pollen allergy sufferers are confronted with longer pollen seasons and pollen with potentially increased allergenicity. The effects for pollen allergy sufferers are an increased duration and severity of symptoms. New research results from the Medical University of Vienna prove that out of the most important air pollution parameters (particulate matter, nitrogen dioxide, sulfur dioxide, and ozone) especially ozone causes increased symptom severity in pollen allergy sufferers during the birch, grass, and ragweed pollen seasons.

## Introduction 

The prevalence of pollen allergy sufferers is increasing in numerous countries [1]. According to studies, between 10 and 30%, i. e., ~ 400 million people worldwide, are affected by allergic rhinitis [2]. This means not only a reduction in the quality of life for a significant proportion of the population, but also an enormous burden on the health system: according to estimates, the direct and indirect costs of an untreated allergy sufferer amount to € 2,400 per year in the European Union (EU) [3]. 

According to numerous experts, air pollution plays an important part in this development [4]. 

## Materials and methods 

### Air quality parameters 

Before comparing air quality data with pollen and symptom data, the effect of air pollution on humans and plants must be understood. Therefore, an extensive literature review was conducted on the major air quality parameters (particulate matter, nitrogen dioxide, sulfur dioxide, and ozone). 

### Influence of air pollution on pollen allergy sufferers 

For this study, the relationship between symptom data, pollen concentration, concentration of individual air quality parameters, temperature, and relative humidity was statistically calculated for the birch, grass, and ragweed seasons in 2010 – 2018 for Vienna. 

Symptom data were obtained from the pollen diary (www.pollentagebuch.at) of the the European Aeroallergen Network. Here, users voluntarily enter their symptom data in an app or on the internet. 

Pollen data come from the Austrian Pollen Warning Service and were measured with a volumetric Hirst-type pollen trap in Vienna [5]. 

Data on air quality parameters were provided by the Municipal Department 22 for Environmental Protection of the City of Vienna. 

Data on temperature and humidity were provided by Zentralanstalt für Meteorologie und Geodynamik (ZAMG; www.zamg.ac.at). 

Statistical analysis was performed using two models. Model A includes the above parameters excluding temperature and humidity. Model B contains the above-mentioned parameters including temperature and humidity. 

### Global warming and air pollution 

The issue of global warming and air pollution has an increasingly strong media presence. Ongoing efforts notwithstanding, temperature and air pollution continue to rise. Both issues are of great concern to pollen allergy sufferers. 

**Figure 1:**
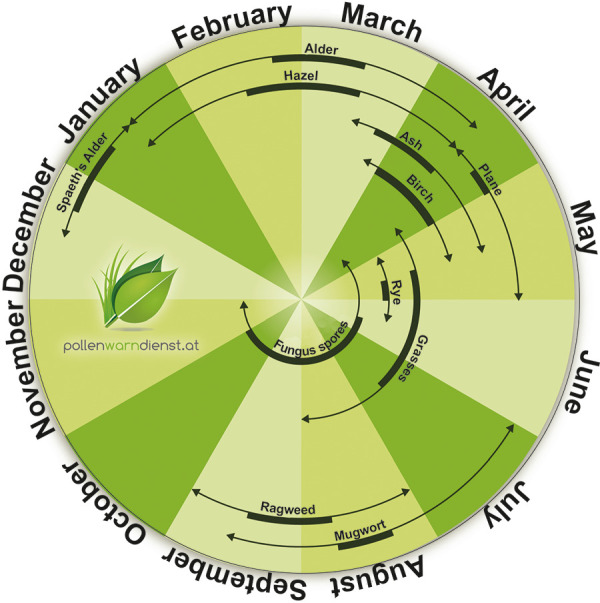
Annual pollen seasons: The black bar represents the peak season (80% of the pollen total for the year); the thinner black arrows represent early and late seasons (10% of the pollen total for the year). **provided by www.pollendienst.at (Medical University Vienna**

Rising temperatures affect pollen production and season length. Today, pollen allergy is no longer only relevant in midsummer, but is already a constant companion for some allergy sufferers. General statements that all pollen seasons are now getting longer cannot be made. On average, however, there is a lengthening or “expansion” of the exposure period for those allergy sufferers who are allergic to tree and grass pollen as well as to herb pollen. The continuing spread of neophytes such as ragweed plays a role in this. For some allergy sufferers, the first strains begin as early as mid-December and do not subside again until October (Figure 1). Thus, pollen allergy sufferers can be confronted with relevant amounts of pollen allergens during 10 months of a year. 

The second important aspect is air pollution. Many studies show that severe air pollutants have adverse health effects, regardless of pre-existing conditions [6, 7]. However, air pollutants not only have an impact on the body, but can also influence the production of pollen and its allergenicity. 

The main air pollutants not only differ in composition but are produced by different processes, and their peak concentrations occur at different times. 

Particulate matter PM_10_: particulate matter is divided into two important categories: PM_10_ (particulate matter) is fine dust < 10 μm. Much of PM_10_ is already trapped in the upper respiratory tract. PM_2.5 _is particulate matter < 2.5 μm. Due to its smaller size, it can penetrate into the lower respiratory tract and is therefore more harmful to health. The point of origin of fine dust is in combustion processes of, for example, heaters (oil and wood) and abrasion (brakes, tires). Forest fires and fireworks also produce large quantities of fine dust. In Central Europe, peak levels of particulate matter occur in winter. Particulate matter mainly affects the cardiopulmonary system and can contribute to the development of lung cancer [8]. 

Nitrogen dioxide (NO_2_): the main cause of NO_2_ production is fossil fuel combustion (car exhaust). Nitrogen dioxide can increase asthma symptoms [9]. In addition, elevated concentrations of NO_2_ can lead to an increase in the allergenicity of pollen [10]. 

Sulfur dioxide (SO_2_): sulfur dioxide is also produced during the combustion of fossil fuels and causes inflammatory processes in the respiratory tract [11]. An influence on the allergenicity of pollen is currently being discussed, but is still unclear [12]. 

Ozone (O_3_): ozone exists in two layers of the atmosphere. In the stratosphere, ozone protects against UV radiation from the sun. In the troposphere (the area where humans live), ozone is a greenhouse gas. This is where the interaction with humans and plants takes place. In large cities, much of the ozone is produced by a reaction between UV radiation and nitrogen oxides. Thus, the peak loads occur in midsummer. In the human organism, elevated ozone concentrations lead to decreased lung function [13]. Exposure of the lungs to elevated concentrations for prolonged periods increases the risk of death from lung-associated disease [14]. Ozone also interacts with pollen and increases its allergenicity. One study also demonstrated that individual ozone molecules can “stick” to the pollen surface and thus also enter the respiratory tract [15]. 

### Ozone as a symptom amplifier 

Our group investigated the influence of individual air quality parameters on pollen allergy sufferers in everyday life [16]. For this purpose, air quality data from Vienna (O_3_, PM_10_, PM_2.5_, NO_2_ and SO_2_) were statistically analyzed with symptom data and pollen concentrations from 2010 to 2018. For this purpose, we used the pollen diary entries of these years during the birch, grass, and ragweed seasons. The results of this study showed that ozone in particular had an impact on the symptom severity of pollen allergy sufferers. Elevated ozone concentrations significantly increased the symptoms of allergic rhinitis in the pollen-allergic patients during the periods of birch, grass, and ragweed pollen flight (model A). In a second statistical analysis (model B), temperature and humidity were included: here, ozone led to increased symptoms in grass pollen allergy sufferers. 

With the seasonal definition used, the 2010 – 2018 birch pollen season had an average duration of 17 days, the grass pollen season a duration of 57 days, and the ragweed pollen season a duration of 33 days. 

We hypothesize that the differences between the results of model A and model B are caused by the longer average season duration of the grass pollen season. Due to the higher number of pollen and symptom data in the grass pollen seasons, statistically significant influences due to air pollution are calculable even when temperature and relative humidity are included. However, it should be noted here that the peak concentrations of ozone in Vienna are mostly measured during the grass flowering season. 

The other air quality parameters analyzed did not show any significant influence on the symptom severity of pollen allergy sufferers. 

However, these results are not intended to show that other air pollutants are harmless to pollen allergy sufferers. In Vienna, nitrogen dioxide and sulfur dioxide concentrations very rarely exceed the threshold value. Particulate matter is definitely measured in elevated concentrations, but the concentration peaks are in winter and thus outside the three analyzed pollen seasons. 

## Outlook 

The study shows that increased air pollution, especially ozone, is relevant for pollen allergy sufferers. 

Pollen allergy sufferers should benefit from the new results. The newly gained information will now be used to further improve the prevention of allergic symptoms for pollen allergy sufferers. The Austrian Pollen Warning Service already includes current ozone concentrations in the personalized forecast model. Access to current ozone information should also give allergy sufferers the opportunity to inform themselves in order to adapt everyday and vacation planning. 

## Conclusion 

**Figure 2:**
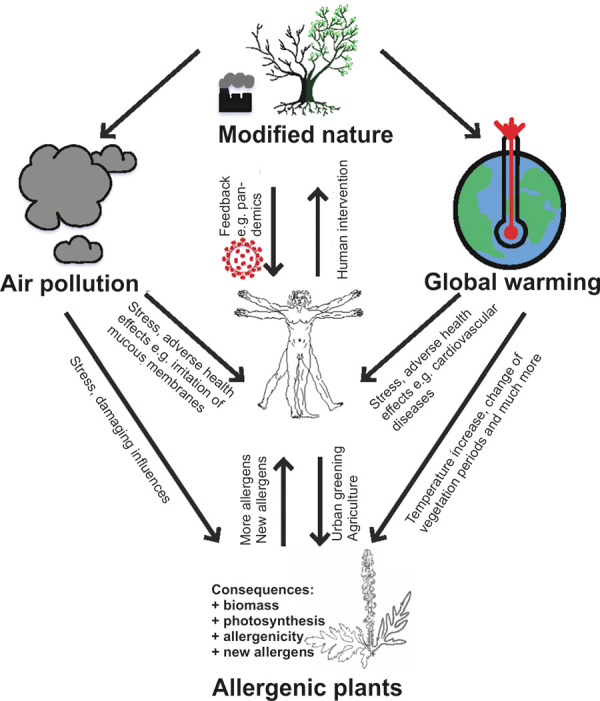
Human-environment interaction.

With the increasing number of reports, it is becoming more and more clear to the population that the effects of global warming and air pollution are already being felt. The current situation is particularly clear for pollen allergy sufferers, as global warming and air pollution interact with both the human body and plants (Figure 2). During the main pollen seasons, ozone in particular causes an increased symptom burden. Therefore, it is important that ozone be implemented in predictive models and that pollen allergy sufferers be better informed. Also, further analysis should be conducted in other major cities with higher air pollutant concentrations to better understand the extent of this situation. 

## Funding 

The statistical analysis and conduction of this study was sponsored by Bencard Allergie GmbH, but the company was not provided with raw data nor had any influence on the research process (study design, collection, analyses, interpretation, writing, decision to submit). 

## Conflict of interest 

No conflict of interest.
